# Management of Asymptomatic Perforation of a Pediatric Rectal Foreign Body into the Peritoneal Cavity Retrieved with Laparoscopy: A Case Report and Review of the Literature

**DOI:** 10.1155/2021/5851967

**Published:** 2021-11-28

**Authors:** Mehdi Forooghi, Hooman Kamran, Reza Shahriarirad

**Affiliations:** ^1^Department of Pediatric Surgery, Nemazee Hospital, Shiraz University of Medical Sciences, Shiraz, Iran; ^2^Student Research Committee, Shiraz University of Medical Sciences, Shiraz, Iran; ^3^Thoracic and Vascular Surgery Research Center, Shiraz University of Medical Sciences, Shiraz, Iran

## Abstract

Rectal foreign body insertion has had an increasing trend throughout the years, whereas it is rarely reported in pediatrics. The management and treatment of these cases can become challenging, since it also can present with atypical or even no symptoms in physical evaluation. A 14-year-old boy was referred to our hospital with a history of insertion of a paintbrush into his anus four weeks before the admission. The paintbrush had perforated the colon and was in the abdominopelvic cavity; however, no symptoms of peritonitis were observed. Rectal examination, sigmoidoscopy, and colonoscopy were unremarkable. Exploratory laparoscopy was performed, and the paintbrush was taken out completely. The patient was discharged in good condition. In cases with rectal foreign body insertion, perforation without causing peritonitis or acute abdomen is possible. In these conditions, imaging examinations play an essential role in managing the patients, and laparoscopy can be a proper procedure for retrieving the foreign body.

## 1. Introduction

Management and treatment of rectal foreign body (FB) insertion are among challenging conditions due to the variety of objects with different shapes and, as a result, different trauma patterns to the rectum and colon [1]. In the examination, our purpose is to obtain information about the FB's size, shape, number, and location [2]. Abdominal pain, rectorrhagia, incontinence, and constipation are the patients' most common complaints [3]. Also, in cases with perforation, peritonitis should be diagnosed early [3]; however, perforation can be presented without any peritoneal symptoms in some patients. Unlike adults, rectal FB insertion in pediatrics is rare and infrequently reported. Herein, we intend to report a 14-year-old boy with a paintbrush inserted into his rectum without any noticeable symptoms in physical evaluation.

## 2. Case Presentation

### 2.1. Chief Complaint and Admission

A 14-year-old boy had been visited by a doctor due to the rectal insertion of a paintbrush, in which computed tomography (CT) of the abdomen was performed and demonstrated a 90 × 6 mm shaped object in the abdominopelvic cavity. He was referred to our hospital for further evaluation.

### 2.2. History Taking and Examinations

**Figure 1:**
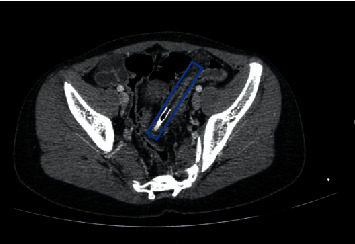
Computed tomography imaging of a 14-year-old boy with evidence of a foreign body (paintbrush) in the pelvic cavity, anterior to sigmoid bowel loops (marked with blue outline).

He reported a history of rectal insertion of a paintbrush four weeks ago, which resulted in rectorrhagia and pain during defecation since that time. The 4-week delay in seeking medical care was because of the patient's mortification. In physical examination, no fever, abdominal tenderness, or rebound was observed. In his CT image, the paintbrush was in the abdominopelvic cavity, located in the middle part of the lower abdomen. Oblique orientation of the FB was seen with the left tip pointing to the left lower abdomen and the right tip to the posterior of the lower abdomen, just anterior to sigmoid bowel loops. The location of the FB was reported in the mesenteric area with adjacent fat stranding and not in the bowel loops (Figure 1). However, the rectal examination was unremarkable. We performed another CT scan for reassurance and reevaluation, which had the same result in comparison to the first image. He underwent rigid sigmoidoscopy under general anesthesia, with no findings in the last 25 cm of the rectum and sigmoid colon. Subsequently, a colonoscopy was performed, which did not show any abnormality. Eventually, exploratory laparoscopy was performed.

### 2.3. Surgical Procedure

**Figure 2:**
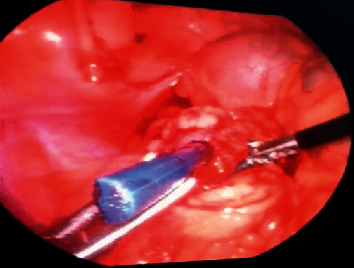
Laparoscopy view demonstrating foreign body (paintbrush) in the omentum.

The patient was taken to the operating room for general anesthesia and was placed in the supine position. Insufflation was done via the insertion of a Veress needle into the umbilicus. Then, a trocar was passed from the umbilicus for the laparoscope, and two laparoscopic graspers were passed from the abdominal wall, one in the right lower quadrant and the other in the left lower quadrant. The peritoneal cavity was explored, and the tip of the paintbrush was found. The paintbrush was covered with omentum, so dissection of the omentum was performed, and the paintbrush was taken entirely out by laparoscopic graspers (Figure 2). No other abnormal finding or intestinal damage was observed in the peritoneal cavity. Finally, the fascia and skin were closed layer by layer.

### 2.4. Postoperation and Follow-Up

The patient received ceftriaxone (25 mg/kg/dose every 12 hours for two days) and metronidazole (10 mg/kg/dose every 8 hours for two days) postoperatively and was discharged in good condition three days after the surgery. The patient has been visited routinely at our clinic postoperatively, with no signs and symptoms presented. After three years from the operation, he is well without any complaints.

## 3. Discussion

The trend of rectal FB insertion is increasing based on studies from western countries [4]. The majority cause of rectal FB insertion is self-/partner-eroticism [4]. Also, it should be noted that criminal assault and psychiatric disorders are two other main reasons for FB insertion [3]. In many rectal FB patients, seeking the medical center may be delayed because of the patients' embarrassment, as in our case [5]. Rectal FB insertion in pediatrics is not as common as that in adults, and fewer studies have been published in this manner. In the present study, we reported a 14-year-old boy with rectal FB insertion retrieved with laparoscopy.

There were two points in our case, which were interesting to be mentioned: (1) the paintbrush had perforated the colon; however, the site of perforation healed, and there was no evidence of any injury in sigmoidoscopy and colonoscopy. (2) Although the paintbrush entered into the peritoneal cavity, there were no symptoms of acute abdomen or peritonitis.

Few studies of pediatric rectal FB insertion are reported in the literature.  Table 1 demonstrates some of these case reports [6–10]. Besides, a study by Fischer et al. [11] reported 27,755 cases, aged 0 to 25 years, with FB injury involving the rectum, vagina, and penis, based on the National Electronic Injury Surveillance System of the United States. The study was performed between 2008 and 2017. In the mentioned study, 51.93% of the cases were from 0 to 18 years that 46.90% of them had rectal involvement. Also, the annual trend of rectal FBs was increasing.

A detailed history and physical examination are crucial in the first step [12]. Physical examination is essential to assess the possibility of peritonitis [13]. In the presence of peritonitis, a perforation is possible, which requires an urgent laparotomy [3]. Rectal examination should be performed as a part of the physical examination to palpate the FB and determine the competency of the anal sphincter [13]. Due to the possibility of inserting a hazardous object (e.g., shattered glass), a detailed history of the item inserted is essential for rectal examination [14]. Some studies suggest the rectal examination to be performed after radiographical evaluation to prevent physicians' injury [5]. Lack of specific symptoms makes the diagnosis and treatment approach more challenging, especially when the patients do not report a detailed history.

Imaging is essential for retained FBs. Although radiography is suggested to be the first diagnostic approach, CT imaging can give more information, especially in the presence of peritoneal symptoms or in higher FBs. Typically, colorectal FBs with perforation can be diagnosed with wall disruption of the bowel and the FB position in the image; however, free gas is not commonly seen. Besides, colovesical fistula or inflammatory mass can be present [15].

There are various therapeutic approaches, including transanal extraction, endoscopic technique, and surgical procedure, for retained FBs [16]. FB should be retrieved with the least possible invasive procedure [3]. Transanal extraction, which is performed with local anesthesia, is successful in 60% to 75% of the cases [17]. If transanal extraction was unsuccessful, an endoscopic approach via rigid or flexible sigmoidoscopy is convenient [3]. The next option is surgery, which can be less invasive with laparoscopy, as in our case, or laparotomy, as the last option [3]. Also, as mentioned, peritonitis shows the possibility of perforation and should be managed with an urgent laparotomy.

**Figure 3:**
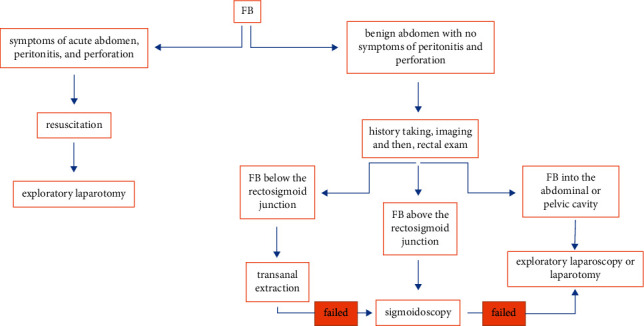
A simplified management algorithm for rectal foreign bodies; FB, foreign body.

There are few reports regarding the management of pediatric rectal FBs in the literature [10]. We propose our simplified management algorithm for rectal FBs (Figure 3). Based on our algorithm, FBs in the gastrointestinal tract cannot be accessed from the abdomen, so transanal extraction/sigmoidoscopy is preferred. However, FBs located in the abdominopelvic cavity are more complicated. According to our approach, when the patient does not present with symptoms of the acute abdomen but has some other symptoms, such as localized tenderness, laparoscopy or laparotomy is suggested after resuscitation. However, in the presence of the symptoms of peritonitis, laparotomy is our choice. In an asymptomatic clinical situation, both laparoscopy and laparotomy can be done. It is worth mentioning that our report addresses FBs that the child has acknowledged the entry of an object into the anus and are so large that they cannot be managed by observation and follow-up, and incidental findings are not included in our report. In our case, we performed laparoscopy because of three reasons: (1) the FB was in the abdominopelvic cavity, (2) the site of perforation healed with no evidence of any injury in the sigmoidoscopy, and (3) the advantages of laparoscopic surgery over open surgery. In an alternative situation, if partial part of the FB had been seen in the sigmoidoscopy, or the rectum wall had not healed, laparotomy would have been our choice.

## 4. Conclusion

In conclusion, our case demonstrated that perforation of the colon and retained FB in the peritoneal cavity may present without acute abdomen or peritoneal symptoms. In these cases, imaging, especially CT scan, can play an essential role in diagnosis and appropriate approach; also, it can be useful for ruling out other conditions. Laparoscopy can be a proper approach in such cases. We suggest a thorough examination before performing any invasive procedure.

## Figures and Tables

**Table 1:** Review of literature regarding rectal foreign bodies in pediatrics.

Author, year	Age (years), sex	Foreign body	Signs and symptoms	Removal procedure/device
Steenvoorde et al., 2003	14, male	Soda can	Abdominal pain, rectal blood loss	Peritoneum clamps
Singer et al., 2012	13, male	Vibrator	Not mentioned	Enemas
Okur et al., 2013	12, male	Empty perfume bottle	Abdominal and anal pain	A blunt clamp
Hamid et al., 2014	5, male	Small pieces of stones	Abdominal pain, constipation	Transanal route, flexible sigmoidoscopy
O'Laughlin et al., 2020	10, male	Wooden golf tee	Diffuse lower abdominal pain	Flexible sigmoidoscopy

## Data Availability

Data of the patient can be requested from the authors. Please write to the corresponding author if you are interested in such data.

## References

[1] Goldberg J. E., Steele S. R. (2010). Rectal foreign bodies. *Surgical Clinics of North America*.

[2] Ayantunde A. A. (2013). Approach to the diagnosis and management of retained rectal foreign bodies: clinical update. *Techniques in Coloproctology*.

[3] Cologne K. G., Ault G. T. (2012). Rectal foreign bodies: what is the current standard?. *Clinics in Colon and Rectal Surgery*.

[4] Ayantunde A. A., Unluer Z. (2016). Increasing trend in retained rectal foreign bodies. *World Journal of Gastrointestinal Surgery*.

[5] Coskun A., Erkan N., Yakan S., Yıldirim M., Cengiz F. (2013). Management of rectal foreign bodies. *World Journal of Emergency Surgery*.

[6] Steenvoorde P., Rovers J. P., Tollenaar R. A. E. M. (2003). Retained foreign body in a 14-year-old boy. *Journal of Pediatric Surgery*.

[7] Singer G., Saxena A. K. (2012). Retrieval of a rectal foreign body using enemas in a 13-year-old boy. *Pediatric Emergency Care*.

[8] Okur M., Kucuk A., Ozkan A., Kaya M., Taskin A. K. (2013). Rectal foreign-body retained by self-sexual stimulation: a case of a 12 year-old boy. *Journal of Academic Emergency Medicine Case Reports*.

[9] Hamid R., Bhat N. A., Wani S. A., Baba A. (2014). Unusual rectal foreign body in a child. *Journal of Pediatric Surgery Case Reports*.

[10] O’Laughlin M., Kim D., Lyman A. L., Lesher A. P. (2020). Pediatric rectal foreign body: value of 3-D CT reconstruction. *Journal of Pediatric Surgery Case Reports*.

[11] Fischer J., Krishnamurthy J., Hansen S., Reeves P. T. (2019). Austere foreign body injuries in children and adolescents: a characterization of penile, rectal, and vaginal injuries presenting to emergency departments in the United States from 2008 to 2017. *Pediatric Emergency Care*.

[12] Kokemohr P., Haeder L., Frömling F. J., Landwehr P., Jähne J. (2017). Surgical management of rectal foreign bodies: a 10-year single-center experience. *Innovative Surgical Sciences*.

[13] Kasotakis G., Roediger L., Mittal S. (2012). Rectal foreign bodies: a case report and review of the literature. *International Journal of Surgery Case Reports*.

[14] Klein E., Bressler M., Nadler S., Shayowitz M., Lapin S. (2020). Rectal foreign body of a shattered glass bottle; case report of unexpected late post-operative hemorrhage managed transanally. *International Journal of Surgery Case Reports*.

[15] Pouli S., Kozana A., Papakitsou I., Daskalogiannaki M., Raissaki M. (2020). Gastrointestinal perforation: clinical and MDCT clues for identification of aetiology. *Insights into Imaging*.

[16] Cawich S. O., Thomas D. A., Mohammed F., Bobb N. J., Williams D., Naraynsingh V. (2016). A management algorithm for retained rectal foreign bodies. *American Journal of Men’s Health*.

[17] Lake J. P., Essani R., Petrone P., Kaiser A. M., Asensio J., Beart R. W. (2004). Management of retained colorectal foreign bodies: predictors of operative intervention. *Diseases of the Colon & Rectum*.

